# Relationship between age and various muscle quality indices in Japanese individuals via bioelectrical impedance analysis (BIA)

**DOI:** 10.1186/s40101-025-00388-5

**Published:** 2025-03-05

**Authors:** Kazushige Oshita, Akihisa Hikita, Ryota Myotsuzono, Yujiro Ishihara

**Affiliations:** 1 Department of Human Information Engineering, Okayama Prefectural University, Soja, Japan; 2 Department of Sport Science, Kyushu Kyoritsu University, Kitakyushu, Japan; 3 Center for Fundamental Education, Okayama University of Science, Okayama, Japan

**Keywords:** Phase angle, Impedance ratio, Resistance ratio, Muscle mass, Skeletal muscle mass index (SMI)

## Abstract

**Background:**

Bioelectrical impedance analysis (BIA) is widely used as a convenient method of measuring body composition. The validity of the phase angle (PhA), impedance rate (IR), and resistance rate (RR) as indices of muscle quality using BIA has been suggested. This study aimed to investigate the relationship between these muscle quality indices and age, and to clarify their characteristics.

**Methods:**

The appendicular muscle mass (AMM), AMM corrected for body mass index (AMM/BMI), PhA, IR, and RR were determined using BIA in 1376 Japanese individuals (532 males and 844 females) aged 15–95 years. The PhA was determined from a 50-kHz current, and the IR and RR were determined from the impedance and resistance ratios between the 250- and 5-kHz currents.

**Results:**

AMM/BMI showed greater age-related changes than the other indices of muscle mass. Significant differences in PhA, IR, and RR were found for the whole body at age ≥ 50 years and for the lower limbs at age ≥ 30 years, compared to those in their 20 s. For the arms, age-related changes were small, and significant differences in PhA of females were only observed at aged ≥ 85 years, whereas significant differences in IR and RR were observed at aged ≥ 75 years, compared to those in their 20s.

**Conclusion:**

These results suggest that although PhA, IR, and RR in the whole body and lower limbs showed age-related changes, the change in PhA in the upper body was small, especially in females. However, IR and RR in the upper limbs of females reflected age-related changes more than PhA.

## Background

The assessment of body composition, including muscle and fat mass, is important for basic health information. For example, the index of appendicular muscle mass (AMM) corrected for height squared or body mass index (BMI) is used as one of the diagnostic criteria for sarcopenia and sarcopenic obesity [1–5]. Further, normal-weight obesity is defined as a state of high body fat despite a BMI within the normal range, and the associated risk of cardiometabolic dysregulation and systemic inflammation has been reported [6–8]. Although body composition can be assessed using various methods, bioelectrical impedance analysis (BIA) is the most frequently used because of the relatively low cost of the basic instrument, ease of operation, and portability [9]. BIA estimates the body composition based on electrical resistance (impedance, Z) by applying a weak alternating current to the body. Although Z comprises the reactance (X) and resistance (R), the X term contributing to the impedance of the body is small, making R equivalent to Z [9]. The resistance of the tissues to electrical currents is directly related to their fluid content. Highly hydrated fat-free mass (FFM) is a good electrical conductor, whereas poorly hydrated adipose tissue is a good electrical insulator [10]. Therefore, this method estimates body composition based on the bioelectrical impedance of the body to the current.

As muscle strength correlates with muscle mass, including muscle volume and cross-sectional area, the bulkier the muscle (that is, the greater its muscle mass), the more tension it can generate and the greater its strength. However, muscle mass does not always reflect the maximum muscle strength, as previously reported. For example, the muscle strength per muscle mass (cross-sectional muscle area) has been reported to be higher in trained individuals than in untrained individuals [11]. Furthermore, although muscle mass declines with the cessation of exercise, muscle strength is maintained or declines at a slower rate than muscle mass [12]. Considering age-related changes, it has been reported that a decrease in muscle mass does not coincide with a decrease in muscle strength [13]. Muscle strength per muscle mass, or muscle quality index [14], has been reported to be associated with life expectancy [15, 16] and physical performance [17]. Therefore, assessing not only muscle mass, but also the contractile capacity of muscle mass, that is, muscle quality, is important.

The essential component of contractile tissue is muscle cells (myofibres); it is important to assess muscle cells (cell mass and/or cell integrity) in addition to total muscle mass (both contractile and non-contractile tissue). Muscle tissue is a collection of muscle cells, and each cell membrane (mainly comprising phospholipids) has a resistive component to the current as a capacitance (that is, this is reflected in X). Therefore, BIA is expressed as the extracellular resistance in parallel with the intracellular resistance, whereas the capacitance of the cells is in series with the intracellular resistance [9]. Based on these electrical properties, the phase angle (PhA) expressed as the arctangent between the R and X, is calculated as (X/R) × (180°/π) and is considered an indicator of cell health, with higher values reflecting greater cellularity, cell membrane integrity, and better cell function [18]. Further, exercise training causes a small decrease in R while significantly increasing X, resulting in an increase in PhA [19]. Because of these characteristics of PhA, an increasing number of recent reports have shown that various physical function indices and physical activity levels have highly positive relationships with PhA [20, 21]. Therefore, the European consensus on the definition and diagnosis of sarcopenia also includes PhA as an indicator of muscle quality [1], and reference values for PhA have recently been proposed for large samples of different age groups [22, 23] or in meta-analyses [24]. Furthermore, other studies have indicated PhA may serve as a robust screening tool for sarcopenia, and cutoff values of PhA for sarcopenia screening have also been provided [25, 26]. However, because these cutoff values may be influenced by ethnicity [25], age-related changes in PhA and PhA values in each age group should be provided for each racial group.

Commercially available BIA body composition analysers use three to six currents: 1, 5, 50, 250, 500, and 1000 kHz [27–31]. Among these frequencies, currents at 50 kHz are commonly used for PhA measurements [32] because currents at approximately 50 kHz have the most significant effect on X. However, this frequency has been reported to average 35.0 kHz or 47.5 kHz for females and 32.6 kHz or 40.2 kHz for males [32, 33], and a study in a young male population found an average of 41.9 kHz with a variation of approximately 10 kHz (standard deviation; SD) [34]. The frequency also varies with age and muscle strength [33]. In other words, the frequency at which X reaches its maximum value is not exactly 50 kHz. Therefore, some studies attempted to assess the muscle quality using a different method via BIA.

The current at 0 Hz, that is, direct current, does not penetrate the cell membranes (the cell membrane has become a perfect capacitor), whereas the cells become transparent to current at ∞ Hz [9]. Therefore, the Z value of the current at 0 Hz is a pure R component and is equal to that of the extracellular compartment. In contrast, Z of the current at ∞ Hz is equal to R for a parallel circuit and is equal to that of the intra- and extracellular compartments [9]. Therefore, when there are fewer intracellular compartment relative to the total tissue mass (intra- and extracellular compartment), the difference in R between the 0- and ∞-Hz currents becomes smaller. In addition, in muscles with a high cell density, the 0-Hz current creates a longer current-carrying distance owing to the increased bypass (that is, higher R), and vice versa in muscles with a low cell density. Previous review articles indicate that the exercise training causes a significantly increasing intracellular compartment [19], and increases in intracellular compartment is associated with improvements in power and strength-performance tasks, independently of weight and lean-soft-tissue changes [35]. Further, the balance between intracellular and extracellular compartments changes with age, and the proportion of extracellular compartment increases [36]. Therefore, evaluating the ratio of intracellular and extracellular compartments to the total tissue using R at 0- and ∞-Hz would be important. In fact, the R ratio between 0- and ∞-Hz has been reported to have a significant relationship between age [33] and might represent differences in physical activity levels that are not reflected in body physique [34]. However, practical constraints and the occurrence of multiple dispersions prevent the use of direct or very high frequency currents [37]. Therefore, Z at low frequency (≤ 50 kHz) currents is considered to mainly reflect the extracellular compartment [38] and Z at high frequency (≥ 200 [39] or 250 kHz [38]) to reflect the intra- and extracellular compartments, and these ratios (called impedance ratios (IRs) [39]) are evaluated as muscle quality. However, as Z comprises R and X, only R (that is, the R ratio of 250- to 5-kHz current) can be used to assess the intra- and extracellular content (refer to as the resistance rate (RR)). Although a relationship with age has been demonstrated for IR [38], a lack of determination of a standardised cutoff has been reported [39]. Furthermore, the RR values for each age group have not been reported.

Thus, PhA, IR, and RR are related but different concepts for assessing muscle quality using the commercially available BIA method. Therefore, the aim of this study was to investigate the relationship between age and PhA, IR, and RR, as well as body composition, using BIA in Japanese individuals and to clarify the characteristics of the relationship between each muscle quality index and age.

## Methods

### Participants

This study included 1376 Japanese (532 males and 844 females), except for 18 participants for whom some data stored in the BIA device were incomplete. Participants were recruited from the attendees of schools, workplaces, and community health screenings, and physical fitness testing events for local residents in two prefectures of Japan. Body composition data were measured using a measuring booth during health checkups and physical fitness events, and the following data were analysed. All participants were informed in writing about the purpose of the study, the content of the measurements (methods, parameters, etc.), anonymity of data use, possibility of withdrawing consent, and their consent for data use was obtained before enrolment. This study was reviewed and approved by the Research Ethics Committee of Kyushu Kyoritsu University (approval number: 2022–08) and Okayama Prefectural University (approval number: 20–72 and 23–62) conducted in accordance with the ethical principles of the Declaration of Helsinki.

The data were collected between December 2020 and December 2024. The inclusion criteria were as follows: (1) (in the case of students) not majoring in physical education or sports science at university (as those who routinely engage in high levels of physical activity are considered to have an unusual body composition); (2) ability to walk independently around the venue; (3) ability to provide informed consent with no evidence of dementia; and (4) not currently using an artificial pacemaker.

### Body physique

Height was measured using a height meter to the nearest 0.1 cm. Body weight was measured using a BIA measuring device attached to the nearest 0.1 kg. Body mass index (BMI) was calculated as weight in kilograms divided by height in meters squared (kg/m^2^).

### Multi-Frequency BIA

A standing 8-electrode multi-frequency BIA (MC-780A-N, TANITA, Tokyo, Japan) was used to measure body composition and bioelectrical impedance. The device measures R and X of the whole body, upper limbs, and lower limbs by applying alternating currents (5 kHz, 50 kHz, and 250 kHz) of less than 90 µA from electrodes on the plantar feet and palms. The participants wiped their palms and plantar surfaces with alcohol-free wet wipes to moisten and clean the electrode contact areas, stepped onto the electrode portion of the machine, and grasped the hand electrodes with both palms for measurement. Participants were asked if they had any urination or bowel movements before the measurement. The participants in the physical fitness event were assessed before the exercise test. All tests were performed in an air-conditioned room between 9 am and 12 pm.

This device measures the PhA in the upper limb, lower limb, and whole body, as well as R and X for each current frequency. The PhA was obtained from the value of the 50-kHz current and evaluated as an absolute value. Z was calculated from R and X, and IR and RR were calculated from Z and R for 5- and 250 kHz currents.

### Body composition

This device measures bioelectrical impedance and the body composition based on it. Body fat percentage (%BF), FFM, upper limb mass (UMM), lower limb mass (LMM), and appendicular muscle mass (AMM) were included in the analysis. In addition, AMM (kg) was calculated as an index divided by the square of the height (m) (that is, skeletal muscle mass index (SMI)) or BMI (kg/m^2^) (AMM/BMI).

### Statistical analyses

Each measure is presented as mean and SD. Further, 5, 25, 50, 75 and 95th percentile values in each age group were also calculated for indices of muscle mass (SMI and AMM/BMI), PhA, IR and RR.

As physical characteristics differed between the sexes, statistical analyses were performed separately for males and females [38]. Each measure was classified as 15–19 years, 20–29 years, 30–49 years, 50–64 years, 65–74 years (young-old), 75–85 years (old), and 85 years and older (old-old), considering the number of participants and the classification of the elderly [40]. One-way analysis of variance (ANOVA) was used to compare the means of the parameters in each age group, and Holm's method was used for multiple comparisons. The StatFlex statistical software (ver. 7.0.10; Artec, Osaka, Japan) was used for these statistical analyses, with a statistical significance level of *P* < 0.05.

Cohen's *d* value was calculated as the effect size for comparing each age group with those in their 20 s. Effect sizes were graded as *d* < 0.2 trivial effect, *d* = 0.2–0.5 small effect, *d* = 0.5–0.8 moderate effect, and 0.8 < *d* large effect.

To examine the association between age and other variables, quadratic regression analyses were performed on the relationship between age and the indices of muscle mass and quality, and the coefficients of determination were determined. Linear regression analyses were also performed on the relationship between PhA, IR, and RR, and the coefficients of determination were determined.

## Results

**Fig. 1:**
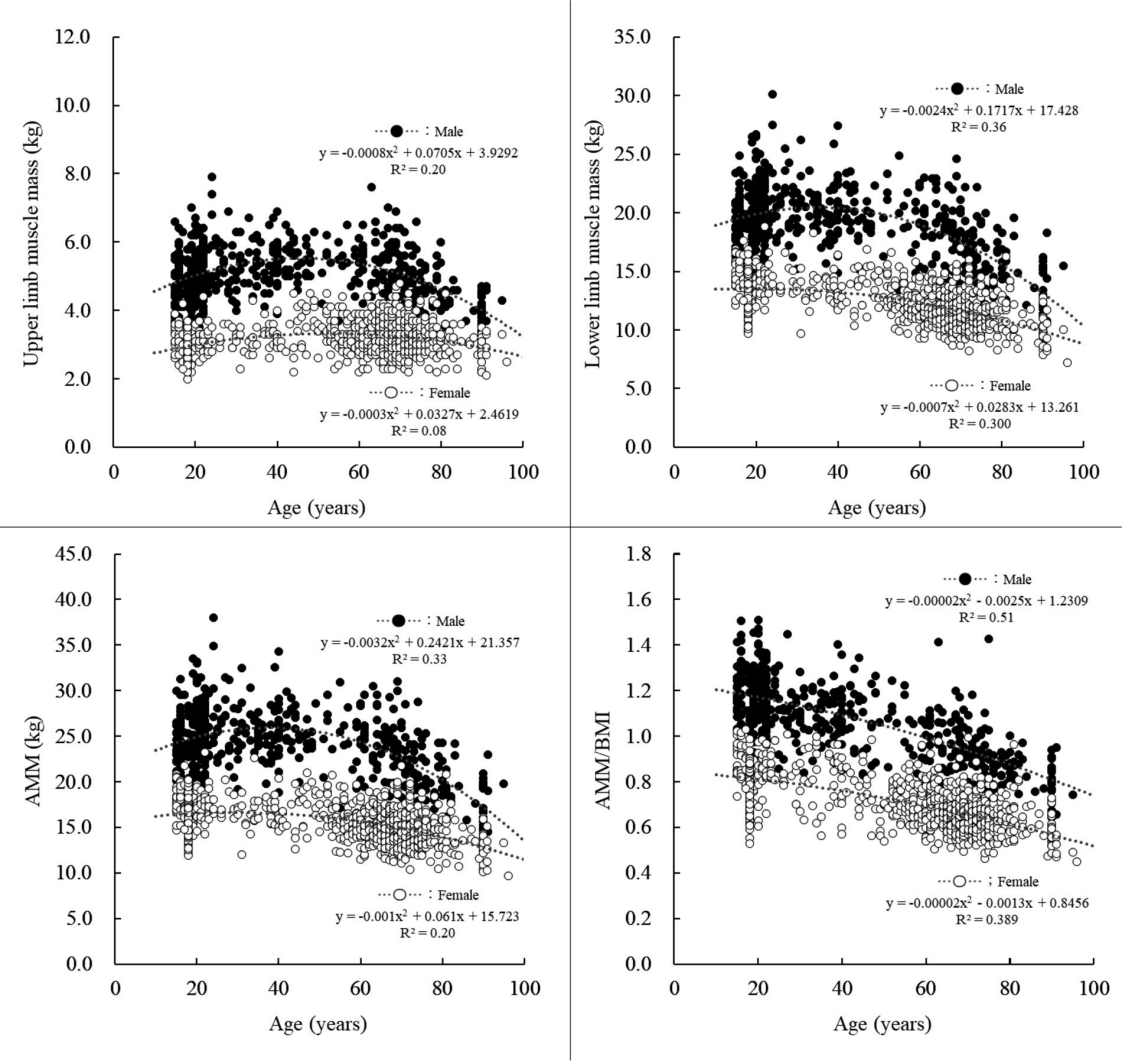
Relationship between age and UMM, LMM, AMM, and AMM/BMI. Closed circles and open circles indicate male and female, respectively. UMM: upper limb muscle mass; LMM: lower limb muscle mass; AMM: appendicular muscle mass; BMI: body mass index

The number of participants in each age group and mean and SD of height, weight, BMI, %BF, FFM, muscle mass, SMI, and AMM/BMI are shown in Table 1, and the relationships between age and muscle mass indices are shown in Fig. 1. For both males and females, significant main effects of age group were found for all indices (Table 1).

**Table 1:** Indices of body physique and muscle mass in each age group

	**15–19 yrs. (a)**		**20–29 yrs. (b)**			**30–49 yrs. (c)**			**50–64 yrs (d)**			**65–74 yrs (e)**			**75–84 yrs. (f)**			**85- yrs**			**ANOVA**
Male																					
N	105		135			97			48			80			42			25			
Age (years)	17.3	(1.6)	22.5	(2.5)		38.8	(5.2)		59.4	(3.9)		72.1	(1.3)		78.6	(2.6)		89.9	(1.0)		
Height (cm)	169.7	(5.6)	172.4	(5.5)	^a^	171.4	(5.4)		170.1	(6.8)		166.1	(5.3)	^bcd^	162.9	(5.7)	^abcde^	159.5	(5.0)	^abcde^	*F* = 30.89, *P* < 0.01
Weight (kg)	58.9	(9.6)	66.5	(9.6)	^a^	68.9	(9.2)	^a^	72.7	(11.2)	^ab^	65.7	(10.0)	^a^	60.5	(9.6)	^bcde^	55.0	(7.2)	^bcde^	*F* = 21.78, *P* < 0.01
BMI (kg/m^2^)	20.4	(2.7)	22.4	(2.8)	^a^	23.5	(2.9)	^a^	25.2	(4.0)	^abc^	23.8	(3.1)	^ab^	22.7	(3.1)	^ad^	21.6	(2.3)	^de^	*F* = 21.16, *P* < 0.01
%BF (%)	13.0	(6.0)	16.2	(5.2)	^a^	17.9	(5.8)	^a^	21.6	(7.8)	^abc^	20.6	(5.6)	^abc^	21.0	(5.9)	^abc^	21.1	(4.5)	^abc^	*F* = 24.08, *P* < 0.01
FFM (kg)	50.9	(6.2)	55.5	(6.6)	^a^	56.2	(5.0)	^a^	56.3	(5.3)	^a^	51.7	(5.0)	^abcd^	47.3	(5.3)	^abcde^	43.2	(3.9)	^abcdef^	*F* = 33.30, *P* < 0.01
UMM (kg)	4.8	(0.7)	5.2	(0.8)	^a^	5.4	(0.6)	^a^	5.5	(0.7)	^a^	5.0	(0.7)	^a^	4.5	(0.6)	^bcde^	4.0	(0.4)	^abcde^	*F* = 24.51, *P* < 0.01
LMM (kg)	19.1	(2.4)	20.6	(2.7)	^a^	20.1	(2.2)	^a^	19.5	(2.5)	^b^	17.0	(2.2)	^abcd^	15.3	(2.4)	^abcde^	13.3	(2.3)	^abcdef^	*F* = 56.16, *P* < 0.01
AMM (kg)	23.9	(3.1)	25.9	(3.3)	^a^	25.5	(2.7)	^a^	24.9	(3.1)		22.0	(2.8)	^bcd^	19.8	(2.9)	^abcde^	17.3	(2.6)	^abcdef^	*F* = 48.26, *P* < 0.01
SMI (kg/m^2^)	8.27	(0.80)	8.69	(0.87)	^a^	8.68	(0.82)	^a^	8.61	(1.03)		7.95	(0.82)	^bcd^	7.45	(0.85)	^abcde^	6.81	(.92)	^abcdef^	*F* = 29.18, *P* < 0.01
AMM/BMI	1.18	(0.13)	1.16	(0.12)		1.09	(0.12)	^ab^	1.00	(0.13)	^abc^	0.93	(0.09)	^abcd^	0.88	(0.11)	^abcde^	0.80	(0.09)	^abcdef^	*F* = 87.67, *P* < 0.01
Female																					
N	129		52			68			163			306			94			32			
Age (years)	17.5	(1.1)	22.8	(2.7)		39.6	(5.4)		58.6	(3.9)		69.3	(2.9)		77.7	(2.6)		89.9	(2.1)		
Height (cm)	156.5	(5.7)	158.9	(5.2)	^a^	157.8	(5.8)		154.7	(5.6)	^abc^	153.3	(5.0)	^abcd^	151.3	(6.0)	^abcde^	147.5	(5.4)	^abcedf^	*F* = 29.46, *P* < 0.01
Weight (kg)	50.0	(7.1)	52.5	(5.8)		54.2	(11.2)	^a^	53.5	(8.5)	^a^	54.8	(8.9)	^a^	51.6	(8.0)	^e^	49.4	(6.8)	^e^	*F* = 6.33, *P* < 0.01
BMI (kg/m^2^)	20.4	(2.4)	20.8	(2.2)		21.8	(4.5)		22.3	(3.3)	^ab^	23.3	(3.4)	^abcd^	22.5	(3.0)	^ab^	22.7	(3.0)	^a^	*F* = 14.45, *P* < 0.01
%BF (%)	27.7	(5.4)	27.4	(5.1)		27.9	(8.7)		29.5	(7.0)		31.6	(7.0)	^abcd^	30.1	(6.8)		31.5	(7.0)		*F* = 8.08, *P* < 0.01
FFM (kg)	35.9	(3.8)	37.9	(2.9)	^a^	38.2	(3.6)	^a^	37.2	(3.3)	^a^	36.9	(3.2)	^acd^	35.7	(3.7)	^bcde^	33.5	(3.1)	^abcdef^	*F* = 11.35, *P* < 0.01
UMM (kg)	2.9	(0.4)	3.2	(0.4)	^a^	3.3	(0.5)	^a^	3.3	(0.5)	^a^	3.3	(0.5)	^a^	3.1	(0.5)	^a^	2.9	(0.4)	^cde^	*F* = 13.81, *P* < 0.01
LMM (kg)	13.2	(1.7)	14.3	(1.3)	^a^	13.5	(1.5)	^b^	12.3	(1.4)	^abc^	11.7	(1.5)	^abcd^	11.1	(1.5)	^abcde^	10.0	(1.3)	^abcdef^	*F* = 61.66, *P* < 0.01
AMM (kg)	16.1	(2.1)	17.4	(1.6)	^a^	16.8	(1.9)	^a^	15.5	(1.8)	^abc^	15.0	(1.9)	^abcd^	14.2	(1.9)	^abcde^	12.9	(1.6)	^abcdef^	*F* = 36.80, *P* < 0.01
SMI (kg/m^2^)	6.56	(0.67)	6.91	(0.53)	^a^	6.75	(0.71)		6.47	(0.68)	^bc^	6.37	(0.68)	^abc^	6.21	(0.63)	^abcd^	5.95	(0.67)	^abcde^	*F* = 12.99, *P* < 0.01
AMM/BMI	0.79	(0.11)	0.85	(0.09)	^a^	0.79	(0.12)	^b^	0.70	(0.09)	^abc^	0.65	(0.07)	^abcd^	0.64	(0.08)	^abcd^	0.58	(0.08)	^abcdef^	*F* = 95.98, *P* < 0.01

Values are expressed as mean (SD). Alphabets indicate significant differences by multiple comparisons

(*P* < 0.05; a vs. 15–19 yrs, b vs. 20–29 yrs, c vs. 30–49 yrs, d vs. 50–64 yrs, e vs. 65–74 yrs, and e vs. 75–84 yrs)

*BMI* body mass index, *%BF*: body fat percentage, *FFM* fat-free mass, *UMM* upper limb muscle mass, *LMM* lower limb muscle mass, *AMM* appendicular muscle mass, *SMI* skeletal muscle mass index

The differences in body physique and composition between the age groups above 30 years and those in their 20 s are described below. The BMI was significantly higher in males and females aged 50–74 years. FFM was significantly lower in males aged ≥ 65 years and in females aged ≥ 75 years, and LMM was significantly lower in males aged ≥ 50 years and in females aged ≥ 30 years. Although the UMM was significantly lower in males aged ≥ 75 years, there was no significant difference in the UMM in females. Table 2 shows the effect size for each group of elderly individuals compared with those in their 20 s. Males showed large sized effects for all age groups, except for UMM for the 65–74 age group, which showed a moderate effect. For females, the effect of UMM was less than moderate, and that of SMI was also moderate up to age 84. AMM/BMI began to be lower in the 30s, and the effect sizes of the elderly group compared to the 20s were greater than those of the other muscle mass indices. These results indicate that (1) LMM and AMM are significantly lower in the middle-aged or young-old groups than those in the 20 s group; (2) UMM is slightly affected by aging (especially in females); and (3) AMM/BMI starts to decline from the age of 30 years.

**Table 2:** Effect sizes (*d*) compared to the 20–29 years group

**Age group**		**Male**			**Female**		
		65–74	75–84	85-	65–74	75–84	85-
**UMM**		0.40	1.02*	1.72*	0.13	0.06	0.55
**LMM**		1.43*	2.04*	2.79*	1.08*	1.34*	2.61*
**AMM**		1.24*	1.87*	2.63*	0.80*	1.08*	2.24*
**SMI**		0.87*	1.43*	2.15*	0.50*	0.69*	1.29*
**AMM/BMI**		2.09*	2.38*	3.00*	1.57*	1.43*	2.48*
**Whole Body**	**PhA**	1.74*	2.63*	3.12*	0.46*	0.77*	1.50*
	**IR**	1.71*	2.69*	3.23*	0.52*	0.84*	1.67*
	**RR**	1.72*	2.69*	3.28*	0.52*	0.85*	1.66*
**Upper limb**	**PhA**	0.64*	1.51*	1.86*	0.05	0.29	0.56*
	**IR**	0.76*	1.62*	2.09*	0.19	0.40*	0.93*
	**RR**	0.75*	1.63*	2.06*	0.18	0.39*	0.85*
**Lower limb**	**PhA**	2.62*	3.37*	4.36*	0.94*	1.32*	2.55*
	**IR**	2.51*	3.31*	4.32*	0.86*	1.23*	2.42*
	**RR**	2.52*	3.32*	4.34*	0.86*	1.22*	2.42*

Asterisk indicates significant differences between the 20–29 years group by multiple comparisons

*UMM* upper limb muscle mass, *LMM* lower limb muscle mass, *AMM* appendicular muscle mass, *SMI* skeletal muscle mass index, *BMI* body mass index, *PhA* Phase angle, *IR* impedance ratio, *RR* Resistance ratio

**Fig. 2:**
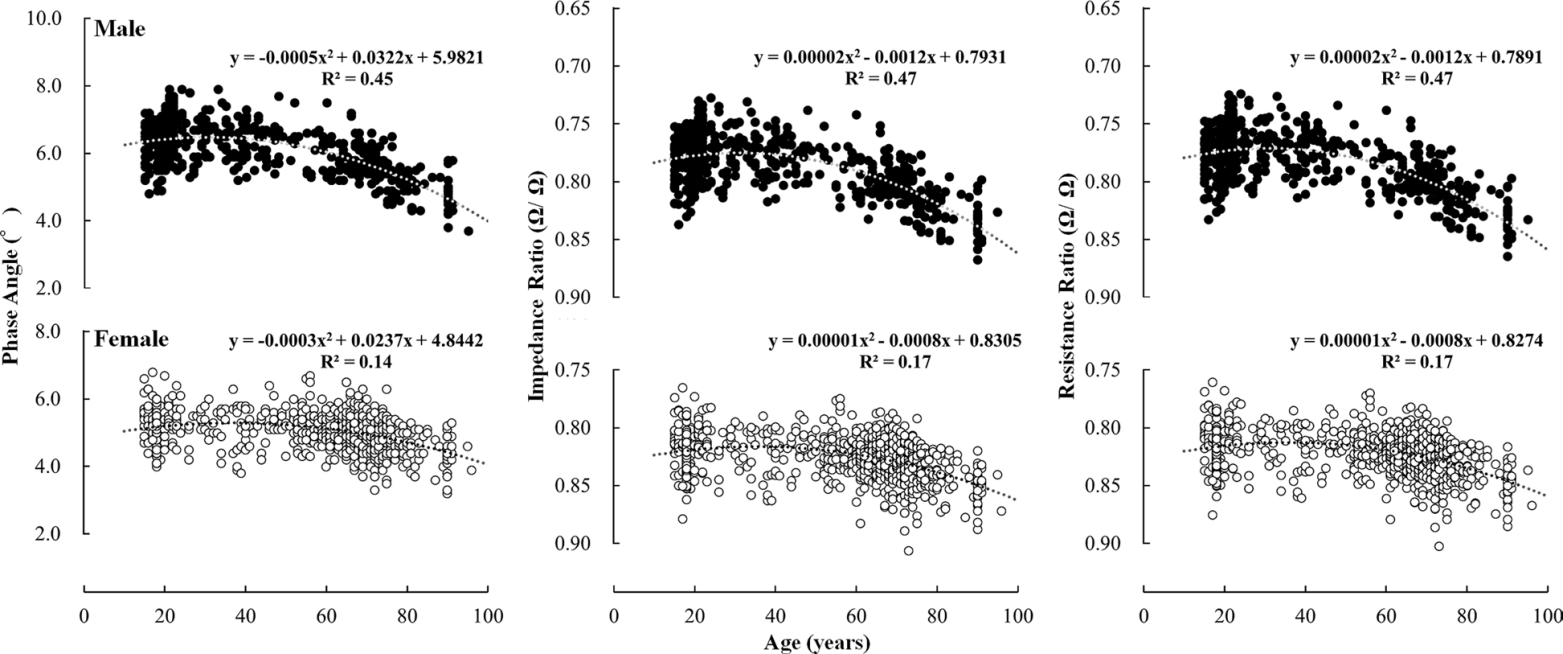
Relationship between age and whole-body PhA (left), IR (centre), and RR (right). Closed circles and open circles indicate male and female, respectively. PhA: Phase angle; IR: impedance ratio; RR: Resistance ratio

**Fig. 3:**
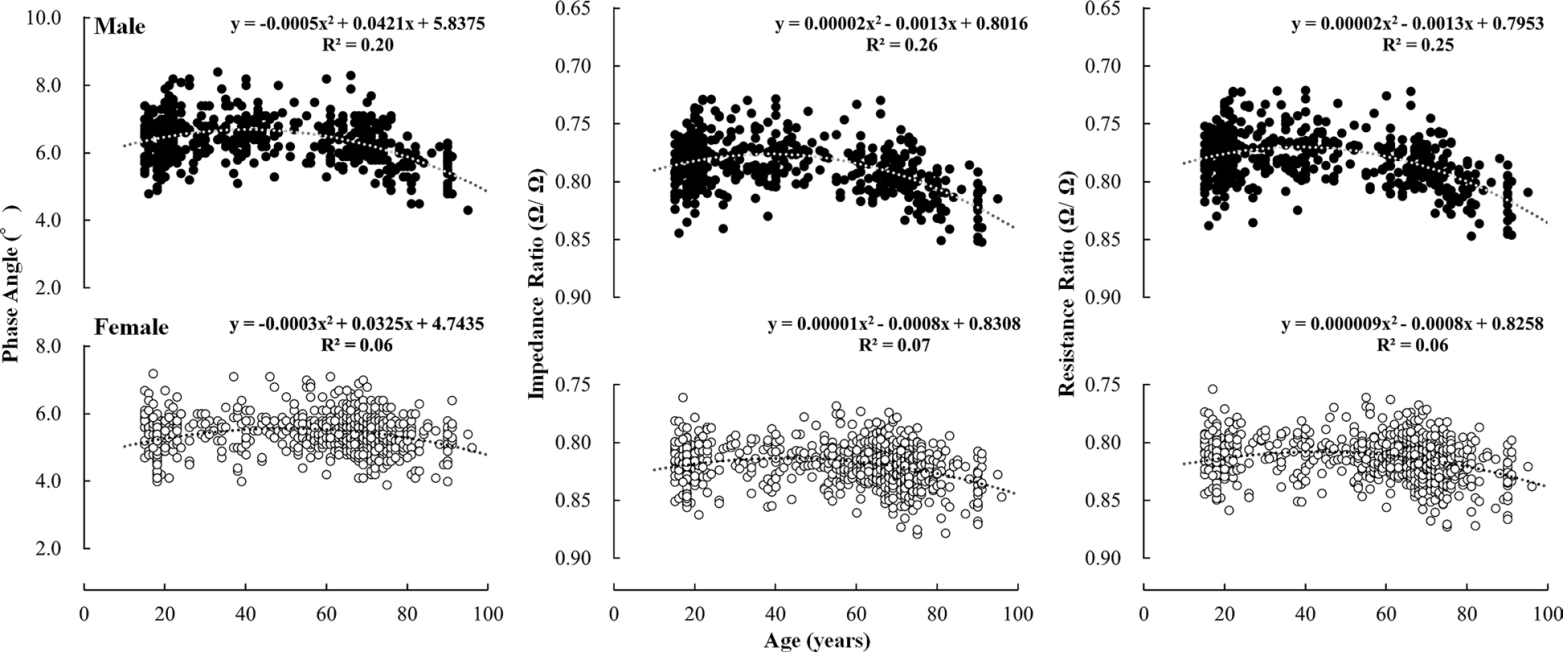
Relationship between age and upper limb PhA (left), IR (centre), and RR (right). Closed circles and open circles indicate male and female, respectively. PhA: Phase angle; IR: impedance ratio; RR: Resistance ratio

**Fig. 4:**
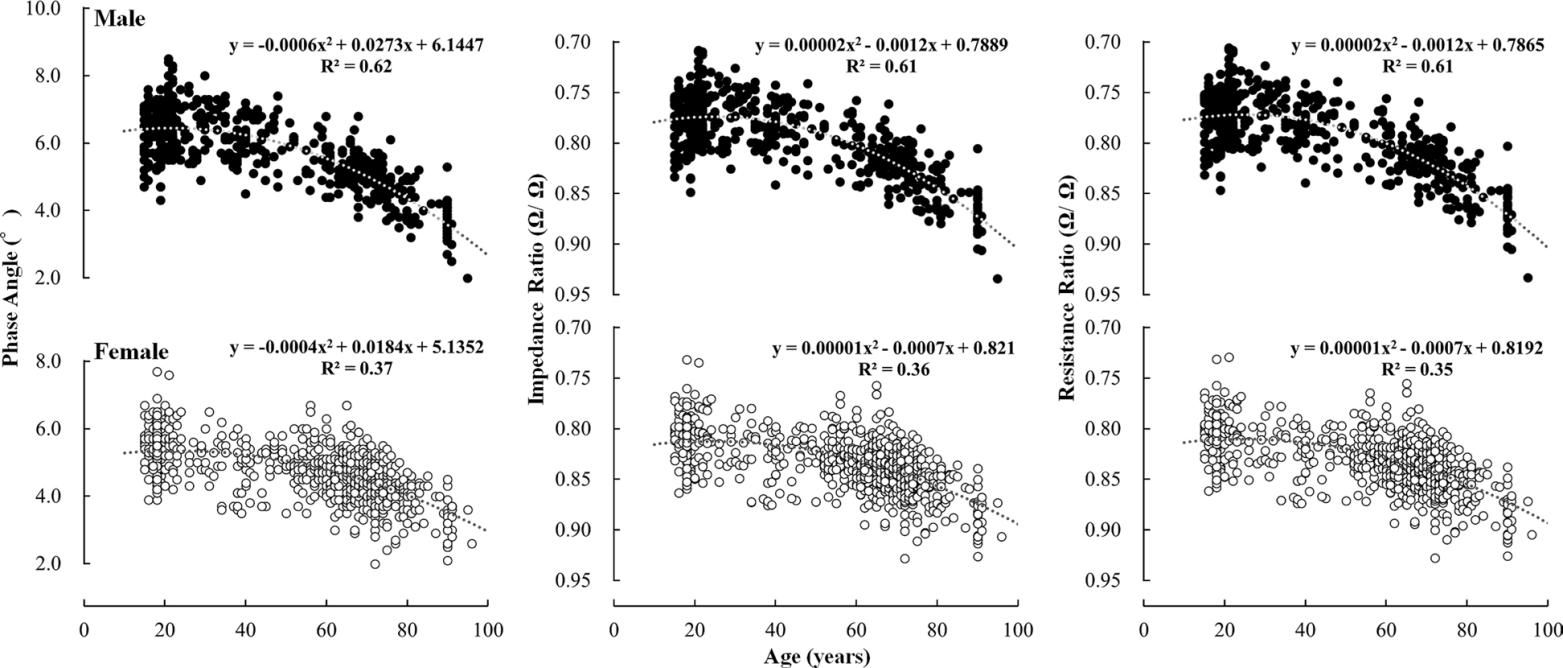
Relationship between age and lower limb PhA (left), IR (centre), and RR (right). Closed circles and open circles indicate male and female, respectively. PhA: Phase angle; IR: impedance ratio; RR: Resistance ratio

The mean ± SD of PhA, IR, and RR for each age group are shown in Table 3, and the 5, 25, 50, 75 and 95th percentile values are shown in Table 4. Further, the relationships between age and PhA, IR, and RR is shown in Figs. 2, 3, 4. For both males and females, significant main effects of age group were found for all indices (Table 3).

**Table 3:** Whole-body, upper limb, and lower limb PhA, IR, and RR in each age group

		**15–19 yrs. (a)**	**20–29 yrs. (b)**		**30–49 yrs. (c)**		**50–64 yrs. (d)**		**65–74 yrs. (e)**		**75–84 yrs. (f)**		**85- yrs**		**ANOVA**
**Male**															
**PhA (deg.)**	**Whole Body**	6.22 ± 0.61	6.56 ± 0.58	^a^	6.44 ± 0.54	^a^	5.98 ± 0.57	^,b,c^	5.67 ± 0.38	^a,b,c^	5.08 ± 0.49	^a,b,c,d,e^	4.84 ± 0.49	^a,b,c,d,e^	*F* = 77.46, *P* < 0.01
	**Upper limb**	6.28 ± 0.61	6.63 ± 0.61	^a^	6.67 ± 0.61	^a^	6.44 ± 0.61		6.26 ± 0.52	^b,c^	5.71 ± 0.59	^a,b,c,d,e^	5.61 ± 0.43	^a,b,c,d,e^	*F* = 25.53, *P* < 0.01
	**Lower limb**	6.23 ± 0.76	6.60 ± 0.69	^a^	6.26 ± 0.67	^b^	5.44 ± 0.63	^a,b,c^	5.03 ± 0.41	^a,b,c,d^	4.35 ± 0.60	^a,b,c,d,e^	3.70 ± 0.62	^a,b,c,d,e,f^	*F* = 143.32, *P* < 0.01
**IR (Ω/Ω)**	**Whole Body**	0.784 ± 0.021	0.773 ± 0.019	^a^	0.775 ± 0.017	^a^	0.793 ± 0.019	^a,b,c^	0.802 ± 0.013	^a,b,c^	0.823 ± 0.016	^a,b,c,d,e^	0.833 ± 0.017	^a,b,c,d,e^	*F* = 81.34, *P* < 0.01
	**Upper limb**	0.787 ± 0.018	0.777 ± 0.021	^a^	0.777 ± 0.019	^a^	0.786 ± 0.019		0.792 ± 0.017	^b,c^	0.810 ± 0.017	^a,b,c,d,e^	0.819 ± 0.017	^a,b,c,d,e^	*F* = 33.02, *P* < 0.01
	**Lower limb**	0.782 ± 0.026	0.769 ± 0.023	^a^	0.778 ± 0.023	^b^	0.806 ± 0.022	^a,b,c^	0.821 ± 0.015	^a,b,c,d^	0.845 ± 0.021	^a,b,c,d,e^	0.867 ± 0.022	^a,b,c,d,e,f^	*F* = 134.80, *P* < 0.01
**RR (Ω/Ω)**	**Whole Body**	0.780 ± 0.021	0.769 ± 0.020	^a^	0.772 ± 0.018	^a^	0.790 ± 0.019	^a,b,c^	0.799 ± 0.013	^a,b,c^	0.820 ± 0.017	^a,b,c,d,e^	0.830 ± 0.017	^a,b,c,d,e^	*F* = 85.53, *P* < 0.01
	**Upper limb**	0.781 ± 0.019	0.771 ± 0.021	^a^	0.771 ± 0.019	^a^	0.780 ± 0.019		0.786 ± 0.017	^b,c^	0.804 ± 0.018	^a,b,c,d,e^	0.812 ± 0.017	^a,b,c,d,e^	*F* = 32.52, *P* < 0.01
	**Lower limb**	0.780 ± 0.026	0.767 ± 0.023	^a^	0.776 ± 0.023	^b^	0.804 ± 0.023	^a,b,c^	0.819 ± 0.015	^a,b,c,d^	0.843 ± 0.021	^a,b,c,d,e^	0.866 ± 0.022	^a,b,c,d,e,f^	*F* = 139.63, *P* < 0.01
**Female**															
**PhA (deg.)**	**Whole Body**	5.12 ± 0.53	5.37 ± 0.49	^a^	5.19 ± 0.56		5.13 ± 0.45	^b^	5.01 ± 0.47	^b,c^	4.75 ± 0.47	^a,b,c,d,e^	4.40 ± 0.53	^a,b,c,d,e,f^	*F* = 21.47, *P* < 0.01
	**Upper limb**	5.13 ± 0.58	5.52 ± 0.50	^a^	5.52 ± 0.57	^a^	5.53 ± 0.50	^a^	5.47 ± 0.51	^a^	5.27 ± 0.50	^c,d,e^	5.15 ± 0.51	^b,c,d,e^	*F* = 11.34, *P* < 0.01
	**Lower limb**	5.34 ± 0.65	5.50 ± 0.69		4.96 ± 0.72	^a,b^	4.80 ± 0.60	^a,b^	4.56 ± 0.59	^a,b,c,d^	4.12 ± 0.56	^a,b,c,d,e^	3.31 ± 0.63	^a,b,c,d,e,f^	*F* = 83.50 *P* < 0.01
**IR (Ω/Ω)**	**Whole Body**	0.820 ± 0.017	0.814 ± 0.017		0.820 ± 0.018		0.823 ± 0.015	^b^	0.827 ± 0.016	^a,b,c,d^	0.837 ± 0.016	^a,b,c,d,e^	0.850 ± 0.017	^a,b,c,d,e,f^	*F* = 29.63, *P* < 0.01
	**Upper limb**	0.820 ± 0.016	0.814 ± 0.018		0.815 ± 0.016		0.817 ± 0.015		0.819 ± 0.016		0.826 ± 0.018	^b,c,d,e^	0.835 ± 0.017	^a,b,c,d,e^	*F* = 9.72, *P* < 0.01
	**Lower limb**	0.813 ± 0.022	0.808 ± 0.024		0.825 ± 0.025	^a,b^	0.830 ± 0.020	^a,b^	0.838 ± 0.021	^a,b,c,d^	0.853 ± 0.020	^a,b,c,d,e^	0.882 ± 0.023	^a,b,c,d,e,f^	*F* = 77.52* P* < 0.01
**RR (Ω/Ω)**	**Whole Body**	0.817 ± 0.018	0.810 ± 0.017	^a^	0.816 ± 0.019		0.819 ± 0.015	^b^	0.824 ± 0.016	^a,b,c,d^	0.833 ± 0.016	^a,b,c,d,e^	0.847 ± 0.017	^a,b,c,d,e,f^	*F* = 29.00, *P* < 0.01
	**Upper limb**	0.815 ± 0.017	0.808 ± 0.018		0.810 ± 0.017		0.811 ± 0.016		0.814 ± 0.017		0.820 ± 0.018	^b,c,d,e^	0.828 ± 0.018	^a,b,c,d,e^	*F* = 8.74, *P* < 0.01
	**Lower limb**	0.811 ± 0.022	0.806 ± 0.025		0.823 ± 0.025	^a,b^	0.828 ± 0.021	^a,b^	0.836 ± 0.021	^a,b,c,d^	0.852 ± 0.020	^a,b,c,d,e^	0.880 ± 0.023	^a,b,c,d,e,f^	*F* = 77.39 *P* < 0.01

Values are expressed as mean ± SD (95% CI). Alphabets indicate significant differences by multiple comparisons

(*P* < 0.05; a vs. 15–19 yrs, b vs. 20–29 yrs, c vs. 30–49 yrs, d vs. 50–64 yrs, e vs. 65–74 yrs, and e vs. 75–84 yrs)

*PhA* Phase angle, *IR* impedance ratio, *RR* Resistance ratio

**Table 4:** 5, 25, 50, 75 and 95th percentile values of SMI, AMM/BMI, PhA, IR and RR in each age group

Age group (years)			Male							Female						
			15–19	20–29	30–49	50–64	65–74	75–84	85-	15–19	20–29	30–49	50–64	65–74	75–84	85-
SMI (kg/m^2^)		95 percentile	9.62	10.09	9.96	10.34	9.52	8.81	7.85	7.89	7.79	8.00	7.90	7.54	7.18	7.24
		75 percentile	8.78	9.27	9.16	9.45	8.65	8.07	7.23	6.96	7.34	7.21	6.85	6.80	6.77	6.12
		50 percentile	8.25	8.76	8.72	8.57	7.96	7.39	6.67	6.45	6.82	6.70	6.33	6.33	6.19	5.93
		25 percentile	7.76	8.07	8.17	7.85	7.48	6.94	6.25	6.02	6.52	6.19	6.06	5.85	5.73	5.51
		5 percentile	6.90	7.19	7.53	7.15	6.91	6.19	5.70	5.73	6.14	5.82	5.64	5.34	5.23	5.06
AMM/BMI (kg/kg/m^2^)		95 percentile	1.37	1.37	1.27	1.21	1.10	1.00	0.95	0.98	0.98	0.96	0.85	0.78	0.79	0.72
		75 percentile	1.27	1.24	1.17	1.08	1.00	0.91	0.86	0.87	0.90	0.88	0.76	0.69	0.68	0.61
		50 percentile	1.19	1.16	1.10	1.00	0.93	0.87	0.80	0.80	0.84	0.81	0.69	0.65	0.63	0.56
		25 percentile	1.09	1.08	1.03	0.91	0.89	0.82	0.76	0.70	0.81	0.70	0.65	0.60	0.58	0.52
		5 percentile	0.97	0.99	0.88	0.79	0.80	0.77	0.66	0.63	0.68	0.57	0.58	0.54	0.53	0.47
PhA (deg.)	Whole Body	95 percentile	7.08	7.60	7.30	6.67	6.60	5.70	5.78	6.00	6.10	5.93	5.80	5.80	5.40	5.15
		75 percentile	6.80	7.00	6.80	6.30	6.00	5.40	5.00	5.50	5.63	5.60	5.40	5.30	5.10	4.83
		50 percentile	6.30	6.50	6.50	6.05	5.70	5.10	4.80	5.00	5.40	5.20	5.10	5.00	4.70	4.40
		25 percentile	5.70	6.20	6.00	5.48	5.50	4.73	4.60	4.80	5.10	4.98	4.80	4.70	4.40	4.10
		5 percentile	5.20	5.60	5.58	5.20	5.20	4.40	4.22	4.30	4.50	4.14	4.41	4.23	4.00	3.47
	Upper limb	95 percentile	7.18	7.63	7.66	7.43	7.12	6.79	6.20	6.10	6.25	6.33	6.50	6.30	6.10	5.75
		75 percentile	6.80	7.00	7.00	6.83	6.63	6.00	5.90	5.60	5.83	5.80	5.80	5.80	5.60	5.50
		50 percentile	6.30	6.60	6.60	6.45	6.20	5.65	5.70	5.10	5.60	5.50	5.50	5.50	5.20	5.15
		25 percentile	5.80	6.20	6.30	5.90	6.00	5.30	5.40	4.70	5.20	5.20	5.20	5.13	5.00	4.90
		5 percentile	5.30	5.70	5.78	5.60	5.80	4.90	4.82	4.30	4.66	4.64	4.90	4.63	4.43	4.32
	Lower limb	95 percentile	7.20	7.83	7.30	6.47	5.81	5.19	4.30	6.40	6.50	5.93	5.70	5.40	4.90	4.39
		75 percentile	6.90	7.00	6.80	5.90	5.40	4.90	4.10	5.70	5.73	5.43	5.10	5.00	4.40	3.63
		50 percentile	6.40	6.60	6.20	5.40	5.20	4.35	3.60	5.30	5.50	5.10	4.80	4.60	4.20	3.20
		25 percentile	5.60	6.15	5.80	4.98	4.80	3.80	3.30	4.80	5.00	4.60	4.50	4.20	3.83	2.88
		5 percentile	5.04	5.50	5.18	4.40	4.30	3.60	2.76	4.20	4.30	3.70	3.81	3.63	3.10	2.50
IR (Ω/Ω)	Whole Body	95 percentile	0.819	0.805	0.801	0.820	0.819	0.848	0.857	0.850	0.845	0.855	0.845	0.855	0.864	0.879
		75 percentile	0.799	0.785	0.788	0.809	0.810	0.832	0.842	0.832	0.822	0.831	0.831	0.837	0.846	0.860
		50 percentile	0.782	0.775	0.773	0.792	0.802	0.822	0.832	0.822	0.814	0.817	0.824	0.826	0.837	0.850
		25 percentile	0.769	0.758	0.766	0.780	0.791	0.814	0.826	0.811	0.803	0.805	0.814	0.817	0.824	0.838
		5 percentile	0.754	0.740	0.748	0.770	0.773	0.796	0.804	0.789	0.784	0.796	0.799	0.801	0.815	0.826
	Upper limb	95 percentile	0.815	0.810	0.803	0.816	0.817	0.833	0.850	0.847	0.846	0.843	0.839	0.847	0.854	0.864
		75 percentile	0.799	0.790	0.790	0.798	0.803	0.822	0.824	0.829	0.826	0.825	0.826	0.830	0.835	0.846
		50 percentile	0.787	0.778	0.776	0.782	0.794	0.811	0.816	0.822	0.813	0.815	0.818	0.819	0.826	0.833
		25 percentile	0.773	0.764	0.768	0.776	0.779	0.799	0.809	0.811	0.802	0.805	0.808	0.809	0.814	0.824
		5 percentile	0.760	0.741	0.740	0.759	0.763	0.782	0.793	0.795	0.788	0.793	0.785	0.792	0.801	0.811
	Lower limb	95 percentile	0.821	0.807	0.816	0.842	0.846	0.873	0.902	0.847	0.849	0.872	0.865	0.870	0.892	0.912
		75 percentile	0.803	0.785	0.793	0.822	0.829	0.865	0.884	0.827	0.824	0.837	0.841	0.852	0.865	0.898
		50 percentile	0.777	0.769	0.779	0.807	0.816	0.845	0.869	0.812	0.807	0.822	0.831	0.838	0.849	0.883
		25 percentile	0.758	0.755	0.760	0.790	0.808	0.826	0.854	0.800	0.800	0.808	0.820	0.825	0.840	0.871
		5 percentile	0.749	0.729	0.744	0.769	0.784	0.812	0.847	0.775	0.772	0.793	0.797	0.809	0.825	0.844
RR (Ω/Ω)	Whole Body	95 percentile	0.815	0.801	0.799	0.816	0.815	0.846	0.853	0.846	0.842	0.852	0.841	0.852	0.861	0.876
		75 percentile	0.796	0.781	0.784	0.806	0.805	0.830	0.838	0.830	0.818	0.827	0.828	0.834	0.843	0.857
		50 percentile	0.779	0.771	0.769	0.788	0.798	0.819	0.828	0.819	0.810	0.814	0.820	0.823	0.833	0.847
		25 percentile	0.764	0.754	0.761	0.777	0.789	0.810	0.822	0.807	0.800	0.801	0.810	0.813	0.821	0.833
		5 percentile	0.749	0.735	0.744	0.766	0.771	0.793	0.804	0.785	0.780	0.792	0.795	0.797	0.811	0.822
	Upper limb	95 percentile	0.809	0.805	0.797	0.810	0.805	0.828	0.844	0.843	0.840	0.837	0.833	0.842	0.849	0.860
		75 percentile	0.792	0.784	0.784	0.793	0.796	0.817	0.819	0.826	0.821	0.819	0.821	0.824	0.830	0.837
		50 percentile	0.780	0.772	0.769	0.775	0.788	0.806	0.810	0.818	0.807	0.808	0.813	0.813	0.820	0.825
		25 percentile	0.767	0.758	0.762	0.769	0.774	0.793	0.802	0.805	0.796	0.799	0.802	0.803	0.809	0.816
		5 percentile	0.753	0.734	0.733	0.752	0.757	0.775	0.787	0.787	0.782	0.788	0.779	0.785	0.794	0.805
	Lower limb	95 percentile	0.819	0.805	0.814	0.840	0.844	0.872	0.900	0.846	0.847	0.870	0.863	0.869	0.890	0.911
		75 percentile	0.801	0.783	0.791	0.820	0.827	0.863	0.882	0.825	0.822	0.835	0.839	0.850	0.863	0.897
		50 percentile	0.775	0.767	0.777	0.805	0.814	0.844	0.868	0.809	0.805	0.820	0.829	0.836	0.848	0.882
		25 percentile	0.755	0.753	0.757	0.788	0.807	0.824	0.852	0.798	0.798	0.806	0.818	0.823	0.838	0.869
		5 percentile	0.746	0.727	0.742	0.767	0.786	0.811	0.845	0.773	0.770	0.791	0.795	0.807	0.823	0.842

*SMI* skeletal muscle mass index, *AMM* appendicular muscle mass, *BMI* body mass index, *PhA* Phase angle, *IR* impedance ratio, *RR* Resistance ratio

The differences in PhA, IR, and RR between the age groups above 30 years and those in their 20 s are described below. In males, significant differences were observed for all indices in the upper limbs over 65 years of age, whereas significant differences were observed in the lower limbs over 30 years of age (PhA was lower, and IR and RR were higher with age). Significant differences were observed in the whole body over 50 years of age. In females, significant differences were observed for all indices over 30 years for the lower limbs and over 50 years for the whole body. For the upper limb, significant differences in PhA were observed only in those aged ≥ 85 years, whereas significant differences in IR and RR were observed in those aged ≥ 75 years. Table 2 shows the effect size for each group of elderly individuals compared with those in their 20 s. In males, all indices showed large effect sizes, except for the upper limb, which showed moderate effect sizes in the 65–74 age group. In females, whole-body PhA had a small effect size in the 65–74 age group and a moderate effect size in the 75–85 age group. Whole body IR and RR had moderate effects in the 65–74 age group. The upper limb PhA had a trivial effect for ages 65–74, a small effect for ages 75–85, and a moderate effect for ages ≥ 85 years. Upper limb IR and RR had small effects for ages 65–85. These results indicate that compared to those in their 20 s, (1) significant differences in PhA, IR, and RR of the lower limbs were observed in those over 30 years; (2) significant differences in the upper limbs were observed in elderly males; (3) although significant differences in IR and RR of the upper limbs were observed in old and old-old females, the effect sizes were not large (in particular, age-related differences in PhA were unlikely to be apparent), and (4) significant differences in whole body PhA, IR, and RR were observed in those over 50 years.

**Fig. 5:**
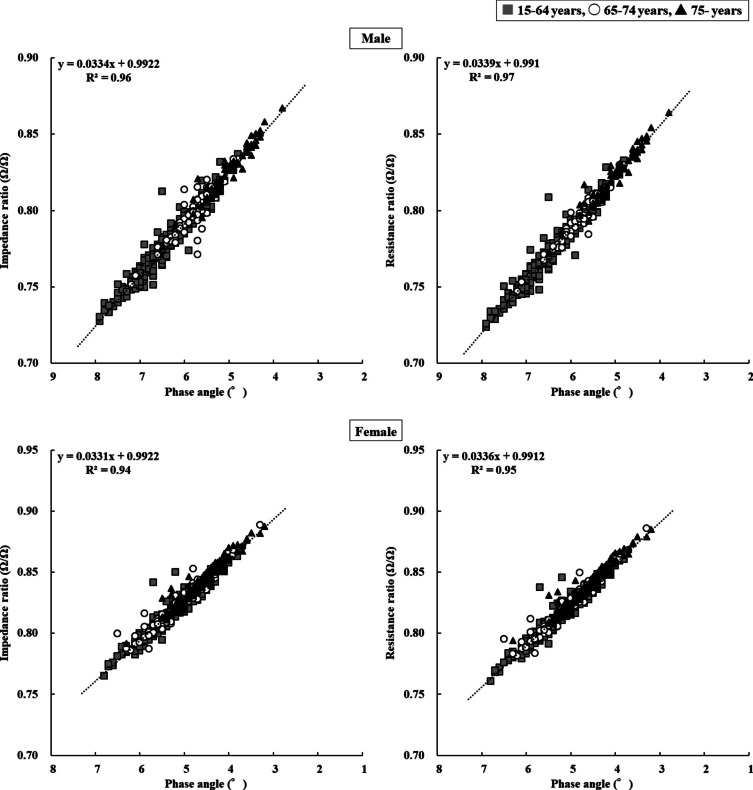
Relationship between whole-body PhA and IR (left) and RR (right). Upper and lower figures indicate values for males and females, respectively. Squares, 15–64 years; circles, 65–74 years; triangles, ≥ 75 years. PhA: Phase angle; IR: impedance ratio; RR: Resistance ratio

The relationships among PhA, IR, and RR are shown in Fig. 5. PhA exhibited a linear relationship with IR and RR, with a coefficient of determination greater than 0.90.

## Discussion

The BMIs of almost all age groups in this study were similar to those reported in the National Health and Nutrition Survey of Japan [41] and other Japanese studies [42], with some fluctuations. Therefore, the participants in this study could be considered to have an approximately normal physique, although only the male group aged 50–64 years had higher values than those reported previously. The results of the National Health and Nutrition Survey of Japan also provide SMI values for people aged over 60 years [41]: (male and female values) 7.8 and 6.5 kg/m^2^ for 64–74 years, 7.2 and 6.2 kg/m^2^ for over 75 years, and 7.0 and 6.1 kg/m^2^ for over 80 years. These values were not greatly different from those of the elderly participants in this study, indicating that they represent standard muscle mass. For site-specific muscle mass, the UMM in the present study did not show a marked age-related decline. Previous studies have also reported that the upper limb loses less muscle mass with age than the lower limb. As common physical activities mainly use the lower body muscles (e.g. walking, climbing stairs), an age-related reduction in activity is thought to be associated with a reduction in lower body muscles [43]. In the present study, the effect sizes for the difference in UMM were smaller in the ≥ 65 years group compared to the 20–29 years group (Table 2) than for the LMM. In particular, the effect sizes (*d*) in females were trivial (~ 0.13) until the age of 84 years and moderate (0.55) in the ≥ 85 years group. A study of 4,003 Japanese participants reported a relatively slow decline in UMM, particularly in females, and the results of the quadratic regression analyses with age showed a coefficient of determination of 0.077 [44], which was almost the same as that of 0.082 in the present study. These results indicate that the population in this study had a body physique and muscle mass similar to those of previous Japanese surveys and research reports.

Because muscle mass is positively correlated with body physique, SMI has long been used as an indicator of muscle mass in the diagnosis of sarcopenia [45]. Further, sarcopenic obesity is defined as the co-existence of sarcopenia and obesity, and the condition can be defined based on values of AMM/ body height^2^ (i.e., SMI), BMI, bodyweight, %BF, and/or waist circumference [4]. A study investigating the association between sarcopenia and fall risk used not only AMM but also AMM/BMI, because AMM/BMI has been reported to be more closely associated with muscle weakness and physical dysfunction than SMI in recently [45]. A more recent study also reported that mobility of middle-aged and older adults correlates with AMM/BMI but not with SMI [46]. Therefore, the foundation for the national institutes of health sarcopenia project recommends AMM/BMI as an indicator of muscle mass in the diagnosis of sarcopenia [3]. Further, AMM/BMI is also a diagnostic parameter for sarcopenic obesity that was recently described in a consensus statement by the Japanese Working Group on Sarcopenic Obesity [5]. In this study, the BMI increased in middle-aged to young-old males and elderly females, whereas %BFs are over 20% in ≥ 50 years male group and over 30% in ≥ 65 years female group. Further, AMM and LMM were significantly lower in the ≥ 65 years male group and in the ≥ 50 years female group, compared to the 20–29 years group. This means that despite an increase or constant BMI, muscle mass decreases (%BF increases). Consequently, AMM/BMI decreased more in the elderly than in muscle mass alone or SMI, as shown by the effect sizes, compared to those in their 20 s (Table 2), and all percentile values between 5 and 95 decrease with age after the age of 30 years (Table 4). Therefore, muscle mass corrected for BMI may be a useful indicator of age-related changes in body composition in the Japanese population.

Significant main effects of age group were found for PhA for the whole body and the upper and lower limbs. Similar to muscle mass, age-related differences were observed in the lower limbs, whereas age-related differences were smaller in the upper limbs. In particular, women's upper limbs did not show significant differences until the old-old group, and the effect sizes were small compared with those in their 20 s. In contrast, the lower limbs showed significantly lower values in the ≥ 30 age group than in those in their 20 s. Whole-body values were significantly lower in the ≥ 50 years age group than those in their 20 s. Several reports have provided reference values for the whole-body PhA. A study of 1967 healthy adults aged 18–94 years found that the PhA was significantly smaller in females than in males and decreased with age [47]. The PhA was (male and female values) 7.9° and 7.0° for 18–20 year olds, 8.0° and 6.9–7.0° for 20–39 year olds, and 6.2° and 5.6° for over 70 year olds [47]. A more recent systematic review reported PhAs of approximately 6.9–7.2° and 6.1–6.3° for ages 19–48, 6.5° and 5.6° for ages 59–69, 5.6° and 5.1° for ages 70–80, and 5.3° and 5.4° for ages over 80 years [24]. Thus, although the values varied between studies, the PhA in this study was lower than those for all age groups. A possible explanation for this could be differences in race. The value of PhA is associated with race [48], and a comparison of PhA by race showed significant differences in the crude analysis: 6.6° for Asians, 6.8° for Caucasians, 7.2° for African Americans, and 7.3° for Hispanics [47]. Although Asians had the lowest PhA, a few Asian samples were included in the aforementioned reference values [24, 47]. Therefore, the PhA in this study may have been lower than those reported in previous studies. The PhAs for the Japanese (male and female) were 6.3° and 5.4° for students aged 18–20 years, 5.3° and 4.6° for the elderly (mean age 73–74 years) [49], 5.3° and 4.7° for the elderly (mean age 72 years) [50], and 5.3° and 4.8° for the elderly (mean ages 75 and 76 years) [51]. These values are similar to those observed in the present study. Therefore, PhA must establish reference values for each race or country.

IR or RR reflects the intracellular compartment relative to the intra- and extracellular compartments, and an IR close to 1 indicates poor cellular health [52] or cellular dysfunction [53]. In this study, IR and RR were found to have a linear relationship with PhA, with a coefficient of determination of 0.90 or higher, which is consistent with previous studies investigating the relationship between IR or RR and PhA [32, 52]. Although IR has been reported to be significantly related to age [38], a lack of a standardised cutoff has been reported [39]. As with PhA, significant main effects of age group were found for IR and RR for the whole body and the upper and lower limbs. Age-related differences were observed in the lower limbs, whereas age-related differences were smaller in the upper limbs. Significant differences in the PhA of the upper limbs of females compared to those in their 20 s were only observed in the old-old group, whereas significant differences in IR and RR were observed in the old and old-old groups. In addition, the effect sizes of the IR and RR for the whole body and upper limbs were larger than those of the PhA for women in their 20 s. Therefore, IR and RR may better reflect age-related changes than PhA as indicators of muscle quality using the BIA method, especially in females. Because this study only compared age with each measurement, the detailed mechanisms are unknown. In other words, because the actual intracellular and extracellular compartments and cell membrane integrity were not measured, the reasons for the differences in PhA, IR, and RR are unknown. Therefore, future studies are needed to clarify the relationship between PhA, IR, and RR and aging by measuring other indicators of muscle quality, and intracellular and extracellular compartments.

This study has several limitations. As mentioned above, this study only examined the relationship between age and other indices; therefore, other indices of muscle quality (for example, muscle strength per muscle mass [14] and ultrasound-derived echo intensity [54]) should be included. Second, this was a cross-sectional study, and age-related changes were unknown. Therefore, a longitudinal study is required to clarify age-related changes. Furthermore, as dietary intake, physical activity, and chronotype affect body composition, including SMI, even in young adults [55–57], further investigations that include different lifestyle habits are required. Finally, there are limitations regarding the sample population of this study. BMI was higher in the male group aged 50–64 years than in previous reports [41, 42]. As PhA is related to BMI, reference values for PhA classified by BMI were provided in a previous study [22]. Therefore, future studies should also provide PhA values for different body physiques. However, a larger sample size is needed to demonstrate this. Although the sample size in this study was 1,376, the studies providing reference values for PhA had larger sample sizes [22, 23, 47]. Furthermore, the data in this study were measured in two prefectures in Japan. Therefore, more data from all parts of Japan should be measured in future studies.

## Conclusions

The aim of this study was to investigate the relationship between age and various indices of muscle quality (PhA, IR, and RR) and body composition by BIA. AMM corrected for BMI was significantly lower in participants aged ≥ 30 years, indicating greater age-related changes. The PhA, IR and RR of the lower limbs were significantly different in those aged ≥ 30 years compared to those in their 20 s. In the upper limbs, age-related changes were small and significant differences in PhA were observed only in the old-old group of females, whereas significant differences in IR and RR were observed in the old and old-old groups. These results suggest that AMM/BMI, as an indicator of muscle mass, may indicate higher age-related changes. PhA, IR, and RR show age-related changes as indices of muscle quality, especially in the lower limbs. IR and RR in the upper limbs of females reflect more age-related changes than PhA.

## Data Availability

No datasets were generated or analysed during the current study.

## Abbreviations

AMM: Appendicular muscle mass
BMI: Body mass index
BIA: Bioelectrical impedance analysis
Z: Impedance
X: Reactance
R: Resistance
FFM: Fat-free mass
PhA: Phase angle
SD: Standard deviation
IR: Impedance ratio
RR: Resistance ratio
%BF: Body fat percentage
UMM: Upper limb muscle mass
LMM: Lower limb muscle mass
SMI: Skeletal muscle mass index
ANOVA: Analysis of variance
